# Supplementary material for: Syncytiotrophoblast Markers Are Downregulated in Placentas from Idiopathic Stillbirths
Source: Int J Mol Sci. 2024 May 9;25(10):5180. doi: 10.3390/ijms25105180 (PMC11121380; doi:10.3390/ijms25105180)
Supplement: Supplementary file 1 [file ijms-25-05180-s001.zip › Supplemental Table S1.pdf]

Table 1: Sample characteristics. The two-tailed Student's t-test was used to statistically compare groups for each variable. Data are given as mean ± standard deviation. iSB: idiopathic stillbirth; SB: stillbirth; LB: live birth.; M: male; F: Female.

Variable	LB (n=3)	SB (n=5)	iSB (n=4)	LB vs SB p value	iSB vs SB p value	iSB vs LB p value
Maternal age (years)	31.333±5.774	30.200±3.834	33.750±7.089	0.746	0.366	0.652
Gestational age (weeks)	37.333±0.577	33.000±4.848	32.250±3.862	0.186	0.809	0.078
