# Supplementary material for: Syncytiotrophoblast Markers Are Downregulated in Placentas from Idiopathic Stillbirths
Source: Int J Mol Sci. 2024 May 9;25(10):5180. doi: 10.3390/ijms25105180 (PMC11121380; doi:10.3390/ijms25105180)
Supplement: Supplementary file 1 [file ijms-25-05180-s001.zip › Supplementary Figures S1 and S2.pdf]

Supplementary Figures

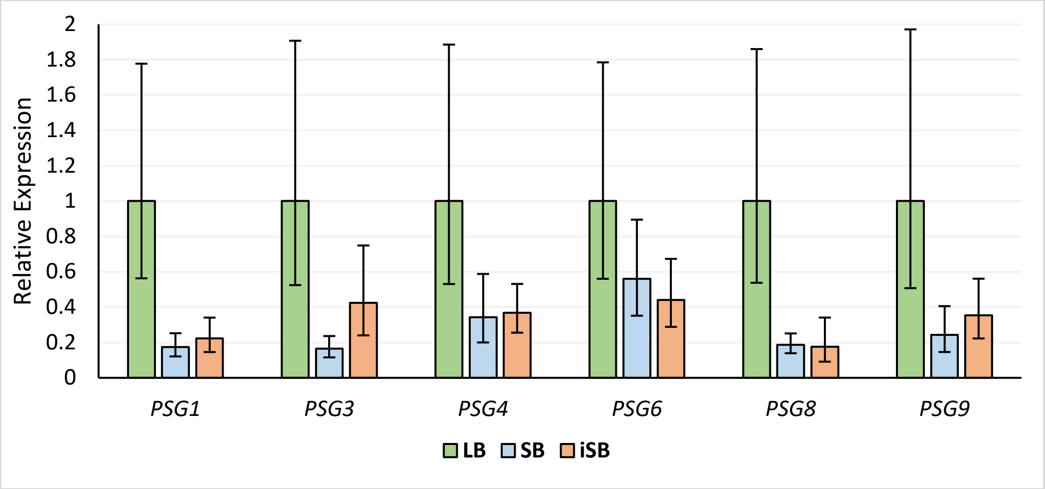

Figure S1: Relative expression of *PSG* genes in placentas from 9 live births (LB), 13 stillbirths (SB) and 12 idiopathic stillbirths (iSB), by real-time qPCR. Three housekeeping genes (*ACTB*, *TBP* and *RPLP0*) were used to normalized the results. Bars represent $2^{\Delta\Delta Ct}$ ± SEM.

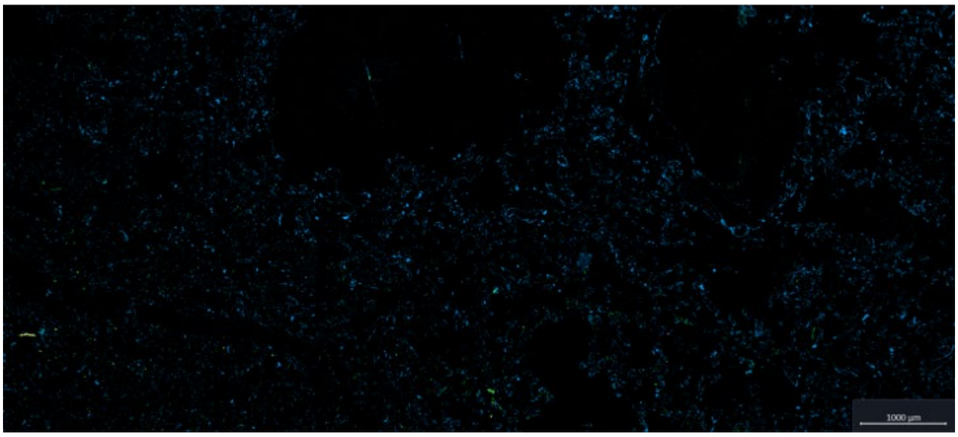

Figure S2: Negative control for Ki67, HLA-G, and COL1 immunofluorescent staining was obtained by omitting the primary antibodies; no signs of fluorescence were detected.
